# Risk factors for Lyme disease resulting from residential exposure amidst emerging *Ixodes scapularis* populations: A neighbourhood-level analysis of Ottawa, Ontario

**DOI:** 10.1371/journal.pone.0290463

**Published:** 2023-08-24

**Authors:** James J. Logan, Amber Gigi Hoi, Michael Sawada, Anders Knudby, Tim Ramsay, Justine I. Blanford, Nicholas H. Ogden, Manisha A. Kulkarni

**Affiliations:** 1 School of Epidemiology and Public Health, University of Ottawa, Ottawa, Ontario, Canada; 2 Department of Geography, Environment and Geomatics, University of Ottawa, Ottawa, Ontario, Canada; 3 Laboratory for Applied Geomatics and GIS Science, Department of Geography, Environment and Geomatics, University of Ottawa, Ottawa, Ontario, Canada; 4 Ottawa Hospital Research Institute, University of Ottawa, Ottawa, Ontario, Canada; 5 Department of Earth Observation Science, Faculty of Geo-Information Science and Earth Observation, University of Twente, Enschede, Netherlands; 6 Public Health Risk Sciences Division, National Microbiology Laboratory, Public Health Agency of Canada, Saint-Hyacinthe, Quebec, Canada; University of North Dakota School of Medicine and Health Sciences, UNITED STATES

## Abstract

Lyme disease is an emerging health threat in Canada due to the continued northward expansion of the main tick vector, *Ixodes scapularis*. It is of particular concern to populations living in expanding peri-urban areas where residential development and municipal climate change response impact neighbourhood structure and composition. The objective of this study was to estimate associations of socio-ecological characteristics with residential Lyme disease risk at the neighbourhood scale. We used Lyme disease case data for 2017–2020 reported for Ottawa, Ontario to determine where patients’ residential property, or elsewhere within their neighbourhood, was the suspected site of tick exposure. Cases meeting this exposure definition (n = 118) were aggregated and linked to neighbourhood boundaries. We calculated landscape characteristics from composited and classified August 2018 PlanetScope satellite imagery. Negative binomial generalized linear models guided by *a priori* hypothesized relationships explored the association between hypothesized interactions of landscape structure and the outcome. Increases in median household income, the number of forest patches, the proportion of forested area, forest edge density, and mean forest patch size were associated with higher residential Lyme disease incidence at the neighbourhood scale, while increases in forest shape complexity and average distance to forest edge were associated with reduced incidence (P<0.001). Among Ottawa neighbourhoods, the combined effect of forest shape complexity and average forest patch size was associated with higher residential Lyme disease incidence (P<0.001). These findings suggest that Lyme disease risk in residential settings is associated with urban design elements. This is particularly relevant in urban centres where local ecological changes may impact the presence of emerging tick populations and how residents interact with tick habitat. Further research into the mechanistic underpinnings of these associations would be an asset to both urban development planning and public health management.

## Introduction

Climate change is having diverse and profound impacts on human health. Extreme heat and erratic precipitation patterns, resulting in issues of food security and increased infectious disease burden are all growing concerns [1]. Governments across all levels recognize the need to respond to these challenges with mitigation and adaptation strategies [2, 3]. In particular, the decisions made by municipal governments can directly address the impacts of climate change at a much more rapid pace than other levels of government [4]. Actions that develop green infrastructure, however, must balance the need to accommodate population growth, through urban expansion by cities of all sizes, and to minimize the resulting impacts on local ecosystems [5, 6]. Intertwined with climate trends and local land use changes are effects on the distribution, life cycles, and transmission dynamics of disease vectors and pathogens. Overall, changes in climate have resulted in an expanded range for many disease vectors in North America and Europe, such as mosquitoes and ticks [7, 8]. At the municipal level, community needs and climate change response strategies–such as urban greening to mitigate urban heat island effects–contribute to changes in the landscape. Such changes often impact local ecosystems in myriad ways. However, efforts to quantify the nature of landscape effects has focused on larger areal units with little analysis of more local vector-borne disease risk [9, 10].

Local transmission of vector-borne diseases is an issue of increasing importance in Canada, attributed to the widening geographic range of disease vectors made possible by the expansion of suitable climatic conditions and habitat [7, 11]. Lyme disease is one of the most significant vector-borne diseases in Canada. *Ixodes scapularis* (blacklegged ticks) transmits *Borrelia burgdorferi* (the bacterial agent of Lyme disease in humans) in eastern North America. Emerging populations of ticks result from the changing migratory patterns of passerine bird species that carry them from regions with established populations (e.g., eastern United States and southern regions of Canada). Once present, ticks may become established under new climatic conditions that allow them to persist through once-inhospitable winters [8]. In 2009, the government of Canada identified Lyme disease as a priority for surveillance, prompting its designation as a nationally notifiable disease [12] and establishing it as an emerging infectious disease of public health concern [13]. In Canada, the incidence of Lyme disease cases has increased from 338 in 2012 to 2,168 in 2022 [14] with cases reported over an expanding geographic range. Active surveillance for questing ticks in the municipal region of Ottawa, Ontario demonstrates that suburban and rural areas of the city exhibit increasingly stable entomological hazard, both in presence of *I*. *scapularis* ticks and *B*. *burgdorferi* infection prevalence among collected ticks [15–17].

In areas where endemicity of tick-borne pathogens, particularly *B*. *burgdorferi*, is long-established, the domestic risk–where exposure to ticks occurs on a residential property–has earned public attention [18, 19]. In the Northeast United States, there is evidence that knowledge of Lyme disease risk has influenced human behaviour, particularly where people choose to live [20]. While awareness of Lyme disease varies across regions of Canada, less than half of Canadians with awareness of Lyme disease risk personally adopt preventive behaviours [21, 22]. Yet, local attention is often required to mitigate environmental Lyme disease risk arising from regional invasions of ticks and the emergence of tick-borne pathogens.

While recent research in Canada has focused on modelling the expected regional presence of the blacklegged tick to inform public health programs [23, 24], there are few evaluations of local impacts where changing municipal landscapes coincide with emergent tick invasions. With few exceptions [25–27], previous studies in eastern North America have focused on Lyme disease risk factors at the regional, state, and county level [28]. Brownstein *et al*. [25] related landscape indices–mean forest patch size and mean patch isolation–to increases in town-level tick density and tick infection prevalence but lower Lyme disease incidence over a twelve-year period in the state of Connecticut. Jackson *et al*. [26] and Seukep *et al*. [27] each examined the relationship between indices of edge contrast–where different land cover types shared adjacency with forested areas–to five-year incidence rates of Lyme disease at a scale comparable to census tracts in the states of Maryland and Virginia, respectively. Both studies found a positive correlation of greater herbaceous-forested landcover edge contrasts with local incidence rates. While the authors of all three studies illuminate the above associations between the interspersion of forests and other land types with peridomestic Lyme disease risk, they do not explore the influence that local ecotones, or how forested land cover is configured and integrated, have within cities. Furthermore, they also assume that all reported cases result from tick bite exposures that occur within the towns or census tracts studied. To address this gap, we simultaneously examined the contributions of neighbourhood-level socio-economic conditions and forested landscape composition to residential Lyme disease risk in a large Canadian population centre. Such research is fundamental for informing landscape management and planning as municipalities grow and adapt.

## Methods

### Study area

**Fig 1:**
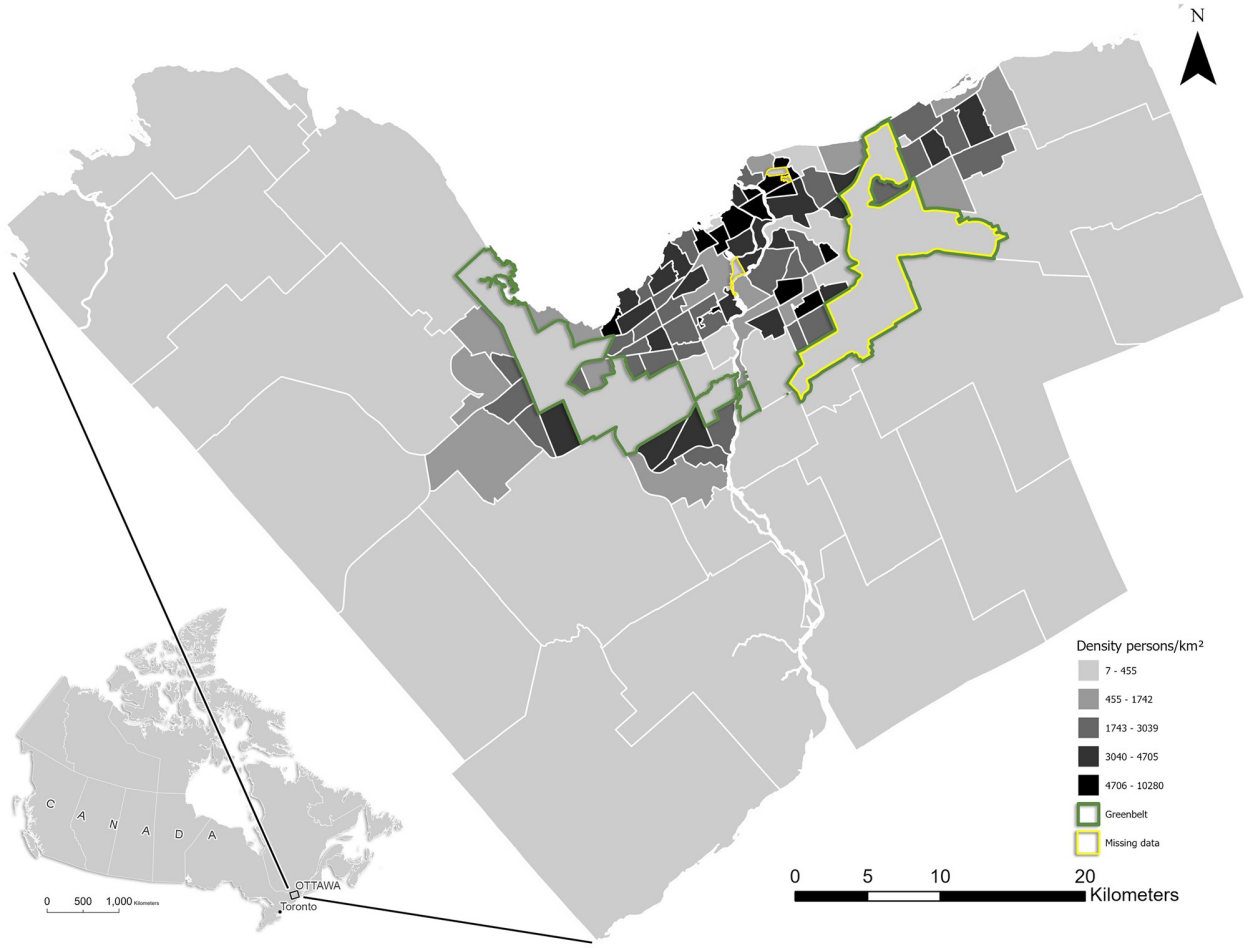
Neighbourhood boundaries (n = 112) with population densities for Ottawa, Ontario. Locations of the Greenbelt and neighbourhoods with no population figures are highlighted.

The City of Ottawa, Ontario extends 45 kilometres south from the Ottawa River and covers nearly 3,000 km^2^ east to west along its shared border with the province of Quebec (Fig 1). Together with Gatineau (the Quebec side of the Ottawa River), it comprises the fourth largest metropolitan area in Canada with nearly 1.4 million people. The City of Ottawa itself contains just over 1 million people inhabiting 112 neighbourhoods within the city limits, as defined by the Ottawa Neighbourhood Study (ONS) [29]. Within these limits, the most densely populated urban areas are partitioned from suburban and rural communities by a protected stretch of trails, forests, farms, and wetlands known as the Greenbelt [30]. This balance of developed and natural environments results in a landscape where residents live within proximity to, and interact with, various habitat types with high biodiversity [6]. We chose neighbourhoods identified by the ONS as the study area unit because they represent ‘natural neighbourhoods’ [31] that have been honed via participatory mapping and community consultation and thus reflect physical and social similarities that affect area residents. Statistical analysis was restricted to 108 neighbourhoods, excluding neighbourhoods with very low recorded population figures (i.e., two cemeteries, one university campus) as well as the expansive Greenbelt, which is aggregated into one neighbourhood in the ONS.

### Ethical approval

We obtained ethical approval for this study from the University of Ottawa Health Sciences and Science Research Ethics Board (file number H06-16-22).

### Dependent variable

The dependent variable in this study is the number of Lyme disease cases in each neighbourhood reported as exposure on an individual’s residential property or nearby neighbourhood locations, adjusted for the total neighbourhood population. We used reported Lyme disease case data between 2017–2020 that were extracted by Ottawa Public Health from the integrated Public Health Information System (iPHIS) based on the Lyme disease case definition [32]. Case records included the age, sex, date of disease onset, address, and up to five reported locations for likely tick exposure, including recent travel history. We mapped cases to residential location by their 6-digit postal code and identified the neighbourhood the case resided in. The first-named exposure location was taken as the most likely and each case was designated as either ‘Residential’, where the provided exposure location matched the patient’s home address exactly or elsewhere within the neighbourhood, or ‘Non-residential’, where the exposure occurred at a location outside the patient’s home neighbourhood. We then aggregated the count of residential cases occurring within home neighbourhoods across all four years of the study.

### Independent variables

We evaluated associations between neighbourhood-level socio-economic and landscape characteristics and the incidence of residential Lyme disease cases. Data sources and methods used to derive each independent variable are described below. The rationale for the use of each measurement in estimating a relationship with the outcome is detailed in Table 1. In all statistical analyses, these independent variables were treated as continuous values and standardized (mean-centred and scaled to units of one standard deviation).

**Table 1:** Rationale and descriptive statistics of variables used to characterize Ottawa neighbourhoods, stratified by presence and absence of residential Lyme disease cases.

			All Neighbourhoods (n = 108)			1+ cases (n = 46)			0 cases (n = 62)		
Variable	Rationale	Units	Mean	Median	Standard Deviation (SD)	Mean	Median	SD	Mean	Median	SD
Proportion forest landcover	High proportions of forested area increase the potential for high abundance of vectors and competent reservoirs.	%	17.41	11.39	18.55	21.26	14.26	20.98	14.62	8.99	16.16
Number of forest patches	Higher numbers of independent forest patches may indicate that more residential properties border forest fragments where small mammal populations can occur in greater abundance [33].	#	34.53	6	64.31	55.47	8.00	81.26	19.34	5.00	43.19
Forest edge-to-area ratio	Forest with more complex geometric shapes (longer perimeter when patches have equal size) allow more residential properties adjacency to forest edge.	km / km^2^	739.35	647.94	390.27	680.2	614.5	369.87	782.3	710.6	401.92
Mean address-edge distance	Neighbourhoods with a shorter distance between addresses and forest edge may indicate more properties border forests, with greater likelihood of tick encounters.	metres	259.71	226.59	174.46	235.87	189.73	181.99	277.02	235.99	168.15
Edge density	Greater amount of forest edge per total neighbourhood area translates to higher amount of other land types bordered by forest, increasing overall opportunities for tick encounters.	km / km^2^	8.70	7.42	6.73	9.24	10.12	5.81	8.31	6.34	7.34
Mean forest patch size	Greater average forest patch size indicates larger forest patches more capable of sustaining a disease transmission cycle with larger small mammal (e.g., white-footed mice) population sizes.	km^2^	2.57	0.13	6.76	3.87	0.41	7.15	1.62	0.08	6.35
Median household income (after tax)	Higher average income is associated with single-detached residential property types [34]. Larger lot sizes may more often border tracts of woodlands and show association with high Lyme disease risk.	$000	$75.74	$76.01	$24.28	$81.10	$82.25	$19.69	$71.84	$73.65	$26.61

#### Ottawa Neighbourhood Study data

The Ottawa Neighbourhood Study (ONS) aggregates and calculates indicators representative of social determinants of health from custom-tabulated Statistics Canada surveys and municipal datasets for all Ottawa neighbourhoods, as explained in detail elsewhere [35]. We selected 2016 median household income (after tax) as a measure of neighbourhood socio-economic status and general measure of inequality to examine its association with incidence of residential Lyme disease [36]. Use of this income variable is consistent with prior studies of Lyme disease risk factors across various geographic scales [26, 27, 37, 38]. The ONS also provided population estimates by neighbourhood generated from the 2016 Statistics Canada census [35].

#### PlanetScope imagery forest measurements

We evaluated the amount of forested area within each neighbourhood using six measurements derived from PlanetScope satellite imagery. To calculate these variables, we selected 38 4-band multispectral images with 3.7-metre spatial resolution taken by PlanetScope Dove Classic satellites during August 2018 and processed to level 3B (orthorectified imagery with a cartographic projection) to capture the extent of vegetation during the summer season [39, 40]. We used images from the final week of this month, selected manually to minimize cloud cover, ensuring all land within the municipal boundaries of Ottawa could be classified. Using Google Earth Engine (GEE) [41], we generated a composite image (mosaic) from the collection of downloaded images to cover the entire city’s extent.

After building the mosaic, we executed supervised classification of land cover using a random forest (RF) classifier in GEE. The RF classifier is a machine learning algorithm that repeatedly fits decision trees (the ‘forest’) to random subsets of a training data set to inform classifications of the broader test data. It is a popular method in remote sensing for its accurate classifications, as well as its ability to handle large amounts of data while being insensitive to multicollinearity between spectral signatures among classes in the provided training samples [42]. We selected training polygons for the classification procedure randomly and equally among five classes: ‘forest’, ‘low vegetation’, ‘built-up / developed’, ‘bare earth / tilled soil’, and ‘water’. Over the entire study area, 400 training polygons (80 per class) covering 1.98 km^2^, approximately 0.1% of the total area, were manually drawn. We used an RF classifier with 30 decision trees to assign each pixel in the composite image to a land cover class, based on the reflectance values imparted from the original multispectral images. We then converted the raster image containing classification data into polygon spatial information with ArcGIS Pro 2.7.2 [43], calculated area and perimeter of all forest patches, and used the Near analysis tool in ArcGIS to calculate the nearest neighbour from each municipal address point to the closest forest patch edge within the City of Ottawa boundaries [44] to represent the degree to which developed land interfaced with woodland.

**Fig 2:**
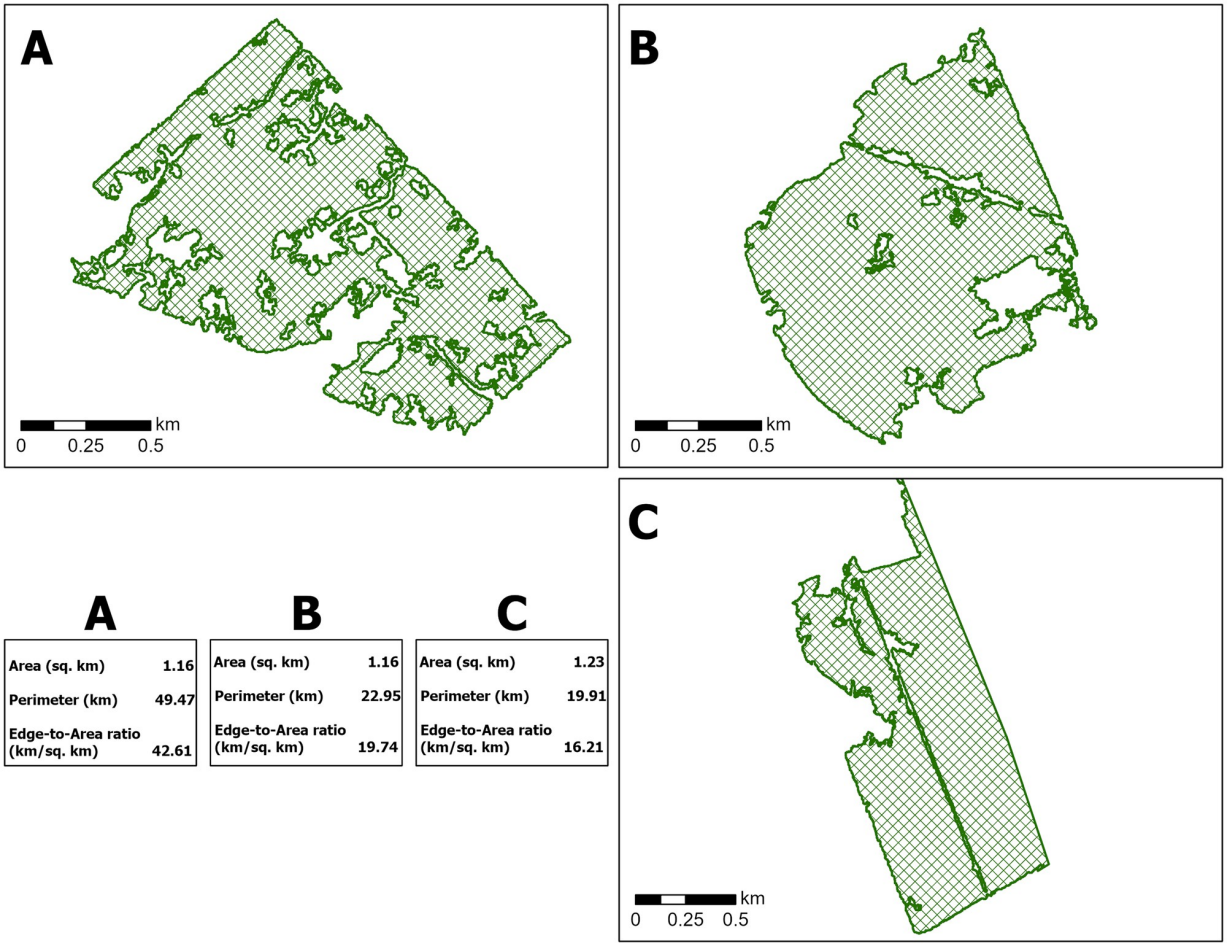
Forest patches of comparable size demonstrating differences in forest shape complexity. Figure was produced with ArcGIS Pro 2.7.2 software and includes the area, perimeter, and edge-to-area ratio of each forest patch depicted.

To account for areas presenting entomological hazard, only forest patches greater than 0.01 km^2^ were included in the subsequent analyses, after Mcclure & Diuk-Wasser and Allan et al. [33, 45] We used a set of landscape fragmentation indices [46] as measures of forest structure and fragmentation in each neighbourhood: the number of forest patches, the mean forest patch size, the proportion of the neighbourhood area that is covered by forest, the edge-to-area ratio (the amount of forest edge, in km/km^2^ of neighbourhood forest area), and forest edge density (the amount of forest edge, in km/km^2^ of the total neighbourhood area). The use of edge-to-area ratio allowed us to compare local forest structures of similar size that exhibit very different shape complexities, as illustrated in Fig 2. In this example, all three forest patches are of comparable size. It is their shapes and, as a result, their perimeters, which are important to consider. In forest patch A, a contiguous forest patch is ‘carved into’ to create residential enclaves that back into the existing woodlands and provide many residents direct access to greenspace. The complexity of the resulting shape is represented by its edge-to-area ratio of 42.6 km/km^2^. By comparison, each of forest patches B and C both exhibit somewhat complex geometries yet have structures that are far less disturbed by the surrounding land use.

### Statistical analysis

For descriptive analysis, we calculated Pearson Chi-square statistics to evaluate significant differences (*α* = 0.05) in means of each variable between neighbourhoods with no residential Lyme disease cases and those with at least one. We considered potential associations between each indicator and residential Lyme disease incidence independently in univariable analyses. We then evaluated selected pairs of these variables, including their interactions, in multivariable analyses. The chosen variable pairs reflect relationships hypothesized *a priori* between the neighbourhood characteristics and the occurrence of Lyme disease from residential exposure, as outlined in Table 2. We calculated Pearson correlation coefficients between pairs of independent variables to uncover any potential collinearity in the hypothesized relationships (S1 Fig). To further ensure collinearity did not influence the models, we used R package *mctest* version 1.3.1 [47] to calculate variance inflation factors for each independent variable in each model in the absence of interaction terms.

**Table 2:** Rationale for modelled relationships between neighbourhood social and ecological characteristics.

Modeled relationship	Variables		Rationale
1	Proportion forested landcover	Mean forest patch size	Areas with high proportion of forest cover but smaller mean forest patches can translate to more non-forested areas bordering woodlands, which presents increased potential for local exposure opportunities compared to where high proportion of forest cover is dominated by large, expansive forests.
2	Number of forest patches	Forest edge-to-area ratio	Higher numbers of geometrically complex forest patches in the terrain may be an indicator of more edge shared with non-forested lands compared to other forest stands, with wider access allowing for potentially greater risk of local tick exposure.
3	Number of forest patches	Mean forest patch size	Higher numbers of forest patches with greater average size in the neighbourhood translate to greater reservoir host populations and suitability of disease transmission cycles.
4	Mean forest patch size	Forest edge-to-area ratio	Neighbourhoods with high average forest patch size and high forest patch shape complexity can sustain high reservoir host populations as well as provide increased opportunities for local tick exposures through more forest edge access.
5	Median household income	Forest edge-to-area ratio	Neighbourhoods with high median household income where larger housing lot sizes border long, complex forest edges contribute to increased opportunities for tick exposure risk.

We used a series of negative binomial (NB) generalized linear models (GLM) fit with a log link to identify socio-ecological factors associated with the occurrence of Lyme disease across Ottawa neighbourhoods. Derived from the Poisson-gamma mixture model, NB is ideal for modeling count-based data that exhibit overdispersion among observations. We chose to use the NB model over Poisson because the variance of the dependent variable was greater than the mean (μ = 1.07, σ^2^ = 6.67). As the Lyme disease count totals across Ottawa neighbourhoods were generally low, we further tested for overdispersion with the Poisson dispersion statistic, calculated as the Pearson chi-square statistic divided by the model’s degrees of freedom, in each univariable GLM. Since dispersion was greater than 1.0 in all cases (*P* > 0.01) it was reasonable to proceed with the standard negative binomial distribution for all models, under the assumption that heterogeneity of observations across neighbourhoods is the cause of overdispersion in the data [48].

Spatial autocorrelation (SA) is a property whereby observed values of a variable between adjacent areas may exhibit more systematic similarity (positive SA) or dissimilarity (negative SA) than would be expected by random chance. When SA is unaccounted for, the chance of a Type I error increases due to violation of the assumption of independence between samples [49]. With the *spdep* package [50] for R 4.0.3, we calculated Moran’s I with a weights matrix based on queen contiguity (i.e. neighbourhoods were adjacent if they shared any edge or corner of their boundaries) and Monte Carlo randomization using 999 simulations to evaluate spatial dependence of the observed number of Lyme disease cases between neighbourhoods. Moran’s I for the count of Lyme disease cases was weak (0.33) but significant (P = 0.001), indicating that neighbourhoods located close to one another tended to exhibit similar incidence (S2 Table). To account for this spatial autocorrelation, we used Moran eigenvector spatial filtering [51] wherein the spatial dependence of the neighbourhood Lyme disease counts (i.e., the weights matrix) was decomposed into eigenvectors and included in GLMs as independent variables that allowed us to filter spatial autocorrelation of the neighbourhood-level observations out of model residuals [52, 53].

We used the *MASS* package version 7.3.55 [54] to fit negative binomial GLMs with the first-order queen’s contiguity Moran’s eigenvector as a spatial filter constructed using the *spatialreg* package version 1.2.6 [51]. For all models, to adjust for variations in population size at the neighbourhood level, we set a constant offset term equal to the natural logarithm of each neighbourhood’s total population estimate from the 2016 Statistics Canada census. We calculated incidence rate ratios (IRRs) and the associated 95% confidence interval for all variables by exponentiating the coefficients from each model and assessed model fits using the Akaike Information Criterion (AIC).

## Results

**Fig 3:**
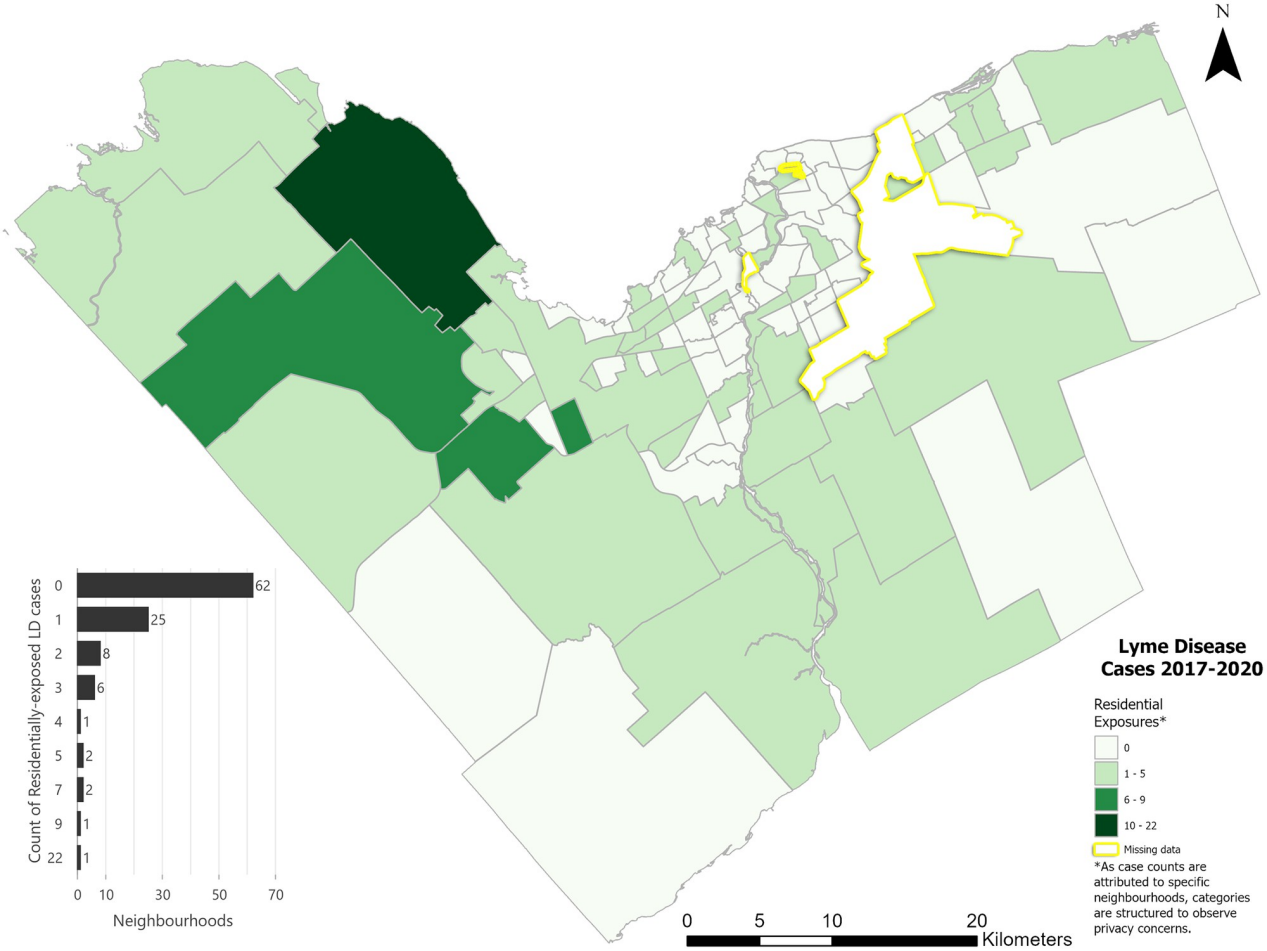
Residential Lyme disease case counts by Ottawa neighbourhoods (2017–2020).

A total of 581 Lyme disease cases were reported to Ottawa Public Health from 2017 to 2020 across the 112 neighbourhoods of Ottawa, Ontario. Forty-four (44) cases did not provide a suspected exposure location and for 3 patients there was no home location available and therefore could not be geocoded. Of the cases with complete spatial information (n = 534; 92%), 427 (80%) named only one location where their tick encounter may have occurred. Exposures that occurred at the patient’s residence or another location within the same neighbourhood accounted for 118 (22%) cases, while 73 cases (14%) cited exposure elsewhere within Ottawa and 343 cases (64%) attributed their exposure to travel outside of the city (S1 Table). Twenty-two (4%) residential Lyme disease cases occurred in one neighbourhood (Dunrobin), whereas 25 (23%) neighbourhoods only had a single residential case (Fig 3). Sixty-two (57%) neighbourhoods, mainly within the Greenbelt area, had no reported Lyme disease cases attributed to residential exposure.

Descriptive statistics for all variables are presented in Table 1. Neighbourhood median household income had a citywide mean of $75,736 (± $24,280 SD) and was higher (p = 0.05) in neighbourhoods with at least one residential Lyme disease case ($81,098) compared to where there were none ($71,844). The RF classifier provided near perfect agreement between the classification and the reference mosaic of PlanetScope imagery (κ = 0.977). Based on its classification result, the highest proportion of forested area among neighbourhoods was 81.7% with a mean of 17.4% (± 18.6% SD). Neighbourhoods where residential Lyme disease cases occurred had just over 6.5% more forest cover on average than those with no cases. The number of forest patches in a single neighbourhood ranged from 0 to 261, while neighbourhoods with at least one case averaged 36 more patches than other neighbourhoods. The mean forest patch size was also larger where residential Lyme disease cases occurred, exceeding the mean size in other neighbourhoods by 2.25 km^2^. Within-neighbourhood ratio of forest edge to area ranged from 0 to 1,778.6 km/km^2^ with a mean of 739.4 km/km^2^ (± 390.3 SD). Neighbourhoods where residential Lyme disease cases occurred averaged 680.2 km/km^2^ in forest edge-to-area compared to 782.3 km/km^2^ among neighbourhoods without cases (P = 0.2). The density of forest edge compared to neighbourhood area was 8.7 km/km^2^ on average (± 6.7 SD), with a lower limit of 0 in neighbourhoods that had no forest patches larger than 0.01 km^2^ and a maximum of 40.8 km/km^2^. Nearest neighbour analysis measuring the shortest distance from each municipal address to the closest point along a forest edge resulted in neighbourhood averages that ranged from 3.8 to 758.1 metres, with a mean nearest neighbour distance of 259.7 (± 174.5 SD) metres among all neighbourhoods.

### Univariable model results

Residential Lyme disease incidence was positively associated with median household income, number of forest patches, proportion of neighbourhood classified as forest, forest edge density, and mean forest patch size (Table 3). Conversely, it was negatively associated with forest edge-to-area ratio and average nearest forest edge distance. These results suggest significant associations between residential Lyme disease incidence and the socio-ecologic composition of the neighbourhoods in which these cases occur. The factor with the strongest positive association is mean forest patch size (IRR: 2.96, p < 0.001), where the model predicts nearly three times higher residential Lyme disease incidence in neighbourhoods where forest patches are one standard deviation (6.76 km^2^) larger on average compared to where they are average size in Ottawa (2.57 km^2^).

**Table 3:** Negative binomial generalized linear model results as incidence risk ratios (IRR) and 95% confidence interval of Lyme disease risk from residential exposure for selected variables.

Variable^a^	IRR estimate (95% confidence interval)	*P*
Median household income (after tax)	2.28 (1.46, 3.67)	< 0.001
Number of forest patches	2.31 (1.75, 3.15)	< 0.001
Forest edge-to-area ratio	0.40 (0.26, 0.58)	< 0.001
Proportion of forested area	2.47 (1.85, 3.37)	< 0.001
Mean address-forest edge distance	0.44 (0.3, 0.62)	< 0.001
Forest edge density	2.35 (1.55, 3.61)	< 0.001
Mean forest patch size	2.96 (2.04, 4.54)	< 0.001

^a^ All variables are standardized; Incidence rate ratio estimates represent the effect of a one standard deviation increase in measurement.

### Multivariable model results

**Fig 4:**
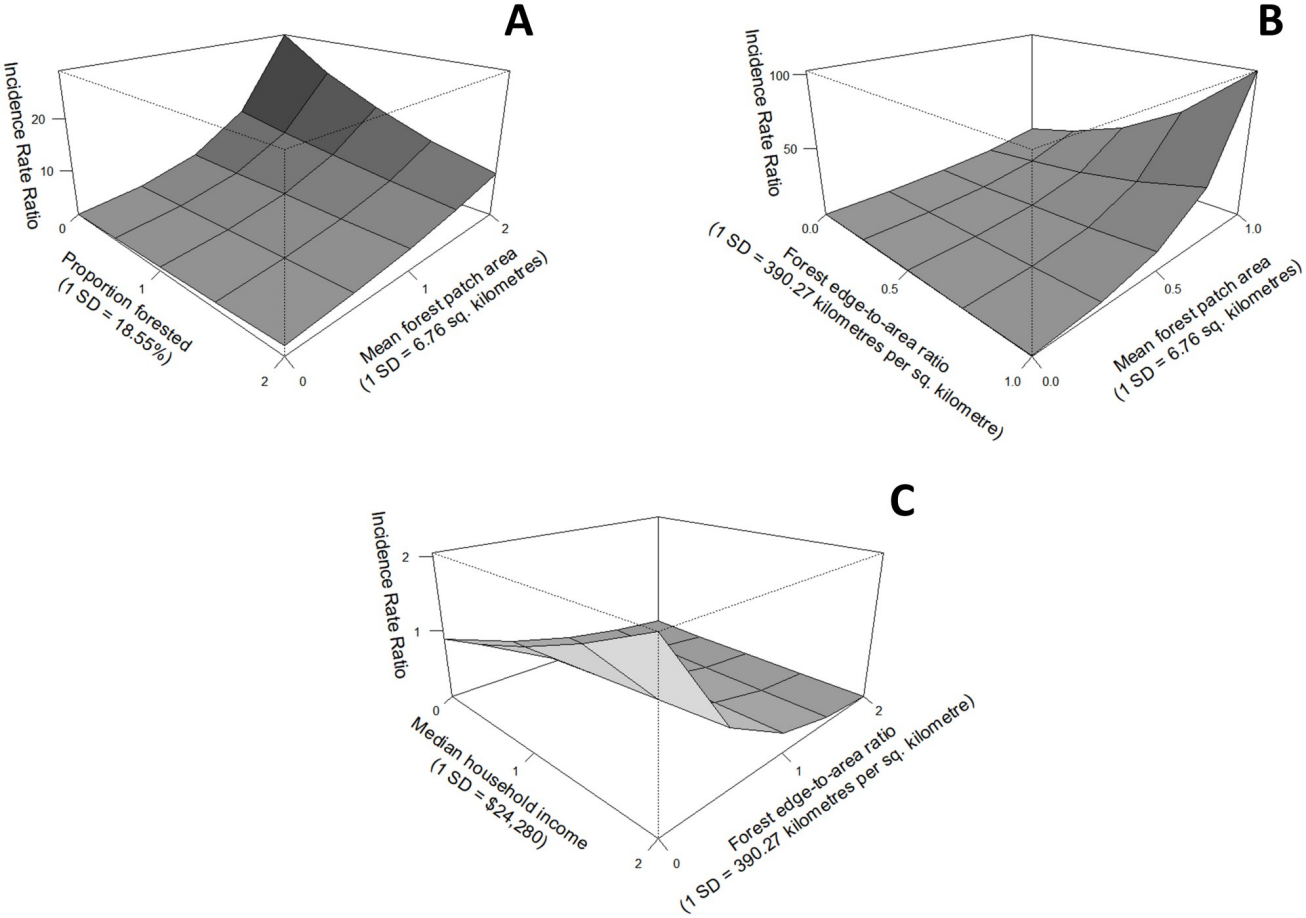
Interaction plots where vertical height represents the incidence rate ratio for the combined effect from changes, in standard deviation units, to proportion of forest cover and mean forest patch size (A), forest edge-to-area ratio and mean forest patch size (B), and median household income and forest edge-to-area ratio (C) in negative binomial GLMs with spatial filtering. The slope along each edge gives each variable’s marginal effect.

Table 4 summarizes results for the hypothesized multivariable relationships between residential Lyme disease incidence and selected variables (detailed in Table 2), both aspatial and with spatial filtering applied. The association of mean forest patch size with predicted residential Lyme disease risk was greatly amplified through interactions with neighbourhood proportion of forested area (model 1) and edge-to-area ratio (model 4), compared with its result in univariable analysis. Interaction terms were statistically significant in three of the five hypothesized relationships (models 1, 4, and 5). We present the interaction plots for these three models in Fig 4 to aid interpretation of the total and marginal effects of the association between each variable and residential Lyme disease incidence. After adjusting for spatial autocorrelation, model 4 performed best as it had the lowest Akaike Information Criterion score (AIC, 252.02), followed closely by model 1 (262.71). Multicollinearity was not detected between individual independent variables by variance inflation factor tests in any of the five modeled relationships.

**Table 4:** Incidence rate ratios and 95% confidence intervals for negative binomial (NB) multivariable generalized linear models, with spatial filtering, of hypothesized neighbourhood characteristic relationships including interaction terms.

Model	Variables^a^	*NB model with spatial filtering*	
		IRR (95% CI)	*P*
1	Proportion forested land cover	1.58 (1.05, 2.36)	0.022
	Mean forest patch size	5.14 (2.49, 11.22)	< 0.001
	Moran eigenvector filter	8.71 (0.51, 153.03)	0.114
	Proportion forest x Forest patch size	0.60 (0.46, 0.77)	< 0.001
	AIC	262.71	
2	Forest patches (> 0.01 km^2^)	1.19 (0.49, 2.89)	0.704
	Edge-to-area ratio	0.53 (0.32, 0.86)	0.013
	Moran eigenvector filter	8.57 (0.28, 246.87)	0.140
	Forest patches x Edge-to-area ratio	0.59 (0.25, 1.37)	0.231
	AIC	273.34	
3	Forest patches (> 0.01 km^2^)	1.77 (1.30, 2.43)	< 0.001
	Mean forest patch size	1.81 (1.19, 2.87)	0.0029
	Moran eigenvector filter	0.01 (0.00, 0.08)	< 0.001
	Forest patches x Forest patch size	0.78 (0.57, 1.07)	0.105
	AIC	258.07	
4	Mean forest patch size	12.51 (4.69, 33.89)	< 0.001
	Edge-to-area ratio	1.38 (0.86, 2.22)	0.179
	Moran eigenvector filter	0.01 (0.00, 0.05)	< 0.001
	Forest patch size x Edge-to-area ratio	4.54 (2.29, 9.16)	< 0.001
	AIC	252.02	
5	Median household income ($000)	1.52 (1.03, 2.24)	0.035
	Edge-to-area ratio	0.41 (0.27, 0.62)	< 0.001
	Moran eigenvector filter	0.00 (0.00, 0.14)	0.003
	Median household income x Edge-to-area ratio	0.59 (0.38, 0.93)	0.022
	AIC	313.45	

^*a*^ All variables are standardized; Incidence rate ratio estimates represent the effect of a one standard deviation increase in measurement.

Univariable analyses of mean forest patch size and edge-to-area ratio demonstrated opposing associations with residential Lyme disease incidence. However, in model 4 (Table 4) these two factors exhibit a significant and synergistic interaction, such that the highest residential Lyme disease incidence was predicted in neighbourhoods with high edge-to-area ratio (forest shape complexity) and large average forest patch sizes (Fig 4B). With this interaction, the association of edge-to-area ratio reversed direction and that of mean neighbourhood forest patch size was greatly amplified. In practical terms, this means that neighbourhoods where greater ecotonal area exists–large woodland areas with peripheries that interface with various land covers (i.e., Fig 2A)–are predicted to experience high Lyme disease incidence. While the independent and marginal effects of these factors were attenuated after adjustment for spatial autocorrelation, their associations remained strong and significant.

In contrast, the marginal effect contributed by the interactions in models 1 and 5 (Table 4) reduced the association of each variable with predicted incidence (Fig 4A & 4C). In model 1, residential Lyme disease incidence is highest when neighbourhood proportion of forested area is low and mean forest patch area is high (Fig 4A). When mean forest patch size is low (or more fragmented) there is a very slight positive relationship between proportion of forested area and incidence. However, with larger mean forest patch sizes (1.5 or more standard deviations above average), the relationship between proportion of forested area and incidence shows a negative trend. Thus, we might expect fewer cases of Lyme disease resulting from residential exposure in neighbourhoods where expansive, contiguous forested areas dominate the landscape compared to neighbourhoods containing one or more large patches but with proportionally lower forest cover. This makes sense as, in the former case, the proportion of developed land would be lower and represent less opportunities in the neighbourhood area for tick encounters.

While statistically significant (p = 0.02), the interaction in model 5 between mean neighbourhood household income and neighbourhood forest edge-to-area ratio exhibited the weakest model fit. Though the model seems to suggest that increases in forest complexity suppress an association between high neighbourhood income levels and residential Lyme disease risk (Fig 4C), the decline in model performance after adjustment for spatial autocorrelation (ΔAIC: 32.46) suggests this should be interpreted with caution.

The number of forest patches in a neighbourhood was the only variable that was not involved in a statistically significant interaction (models 2 and 3, Table 4). In each of these models the marginal effect of interaction terms appears to suppress the effects of each independent variable alone. In model 2, greater numbers of forest patches in a neighbourhood show a slight association with increased residential Lyme disease incidence, which is further dampened as forest shapes increase in complexity. By comparison, the positive association between number and size of neighbourhood forest patches with residential Lyme disease incidence in model 3 are stronger than a negligible interaction effect. In this case neighbourhoods with many forest patches, large woodland areas, or both are predicted to have higher Lyme disease incidence from local exposures. This suggests that while the number and size of these forest patches are strongly and independently associated with residential Lyme disease incidence in Ottawa neighbourhoods, their combined effect is less evident.

## Discussion

This study provides the first assessment of neighbourhood-level socio-ecological characteristics associated with residential Lyme disease risk in a major Canadian population centre. Across the entire study period (2017–2020), just over one-fifth of Lyme disease cases reported to Ottawa Public Health believed they were exposed on their own property or elsewhere in the neighbourhood where they lived. While most Ottawa cases during this period were not considered residential per our definition–other suspected exposure locations include natural areas outside the patient’s home neighbourhood, out to the broader eastern Ontario region, or further abroad–it is important to note that residential Lyme disease exposure may increase in importance with ongoing climate and land use change. Observations of ticks in gardens and mown lawns in areas with established tick populations demonstrate that occurrence of “backyard” encounters may increase [55, 56]. This trend has two important drivers. First, the emergence and establishment of tick populations in new locations in Canada has been demonstrated through surveillance and is likely to continue in northern latitudes [11, 17]. Second, between urban area growth and infrastructure development, the interface between inhabited and natural landscapes will become more intertwined. Thus, it is important to identify characteristics of the urban landscape that may be relevant to public health planning for tick-borne diseases at a local level and consider them in zoning and official plans. A novel aspect of our analysis is the examination of statistical interactions between pairs of neighbourhood forested area measurements and the incidence of residential Lyme disease at the neighbourhood scale. The modeled interactions provided additional insights into the associations between forest fragmentation, forest shape complexity, and residential Lyme disease risk compared with when these factors were considered independently.

We showed that higher Lyme disease incidence is associated with greater forest patch size within local neighbourhoods (Table 3), consistent with what was previously observed at the level of municipalities [25]. In neighbourhoods where the average forest patch size is just over 9 km^2^, there was a nearly threefold increase in the incidence of residential Lyme disease compared to neighbourhoods where forest patches averaged roughly 2.5 square kilometres in size. While Allan et al. [33] demonstrated that dramatically higher Lyme disease risk may result from decreasing forest patch size, their focus was on whether higher entomological hazard posed by increased density of infected nymphal ticks was present in smaller compared to larger forest patches. Our studies are not directly comparable as the response variables and scale of analyses are different. Statistical results from our analysis of cases are dependent on the spatial scale and zonal configuration of the ONS neighbourhoods within which the cases are aggregated. Such differences are induced by the Modifiable Areal Unit Problem in geographic analysis [57] and should be seen as a scale-dependent analysis that is different from that of Allan et al. Where they identify the association between tick hazard present in individual forest patches and the size of those patches, we evaluated residential Lyme disease incidence associated with neighbourhood-mean patch sizes. Furthermore, our multivariable analysis revealed that the effect of mean forest patch size was significantly amplified through the effects of forested land cover proportion or forest shape complexity (edge-to-area ratio) within neighbourhoods (models 1 and 4). Our results suggest that greater average patch sizes have a pronounced association with residential Lyme disease risk when the overall proportion of forested area in the neighbourhood is less than twenty percent. Since our exposure criteria was limited to encounters on patients’ properties or elsewhere in the same neighbourhood, our results highlight that careful consideration of how woodlands and larger forest patches are integrated into the neighbourhood landscape, as well as how they are used by nearby residents, should be exercised.

Our analysis of forest edge-to-area ratio also showed an association with predicted incidence of residential Lyme disease cases, supporting prior findings of a negative correlation between specific landscape edge definitions and Lyme disease incidence at the county level in the United States [27]. A one standard deviation increase (390 km/km^2^) of this ratio above the mean was associated with 60% fewer cases compared to neighbourhoods with Ottawa-average forest complexity. However, analysis of the combined effect of edge-to-area ratio with average forest patch size (model 4, Table 4) allowed us to examine the effect of forest complexity disentangled from forest area by holding average patch size constant. This revealed a more complex relationship of landscape designs with high edge-to-area ratio. Where development encroaches on larger forest patches, increasing forest edge and, presumably, the risk for human tick encounters in the neighbourhood at those edges, the interaction model predicted higher Lyme disease incidence resulting from local tick exposures. Fig 2A illustrates one hypothetical example of this–a large woodland that is carved up but not fragmented, allowing more residential properties and additional opportunities for exposure through new woodland interface. By comparison, Fig 2B and 2C depict forest patches of similar size to Fig 2A but with much smaller edge-to-area ratios, which is associated with lower residential Lyme disease risk according to Model 4 results. Though our analysis does not specify the types of edge contrast (e.g., forest-low vegetation, forest-developed) in our estimation of forest edge measurement, the general effect of edge within neighbourhood areal units is significant, indicating greenspace that exhibits complex shapes and its integration with other landscape types merits further exploration–particularly in developed residential areas where tick populations are established.

Our results also suggest that higher Lyme disease incidence occurs in wealthier neighbourhoods. In our univariable analyses, a neighbourhood with a median after-tax household income of approximately one hundred thousand dollars per year had more than twice as many cases of residential Lyme disease than neighbourhoods with the mean income level ($75,736). This is consistent with previous studies, which associated higher socio-economic levels with greater Lyme disease risk regardless of where the tick encounter occurred [58]. Unexpectedly, our interaction model evaluating household income and forest edge-to-area ratio revealed a negative association of their combined effect with the incidence of residential Lyme disease cases. When neighbourhoods exhibit a city-average level of forest shape complexity (e.g., 739.35 km/km^2^), increases to neighbourhood median household income result in higher Lyme disease incidence. One interpretation for this result is that in wealthier peri-urban communities, where large residential lots may border tracts of woodland, Lyme disease risk is higher. Yet, more complex neighbourhood forest structures drastically reduce the effect contributed by neighbourhood socio-economic levels–counter to the relationship hypothesized *a priori*. It is possible that forests with greater edge occur in areas with more residential properties and thus smaller lot sizes, which is not captured by the aggregated nature of these data. Property-level effects and resident behaviours (e.g., lawn maintenance, adoption of preventive measures) could further confound a potential relationship in neighbourhoods characterized by high socio-economic levels and complex forest structures. Higher socioeconomic status also generally confers increased access and use of healthcare services [59] and it is possible that healthcare access inequality might confound the observed effects due to differences within or between neighbourhoods. Furthermore, this model exhibited a lack of fit and residual spatial autocorrelation after spatial filters were applied, suggesting that while the combination of these values was significant in our study location it was not fully independent of the underlying spatial process or the definition of our variables at the chosen level of aggregation. Additional research at increased resolutions–focusing on details at the residential property level–could be helpful to identify the effects involved.

These results indicate a significant association with residential exposure risk in more heavily forested neighbourhoods with greater levels of forest shape complexity, or where large patches of forest are interspersed with and broken up by developed land. It is also true of wealthier peri-urban communities, where, in cities such as Ottawa, such neighbourhoods are comprised mainly of single-family homes, often situated on lots bordering tracts of woodland. For urban centres like Ottawa, where residential expansion into woodland areas is an alternative to intensification, this emphasizes that community design and structure are connected to local Lyme disease risk. Thus, community landscape composition warrants consideration in vital public health risk assessment. Urban development and greening initiatives will produce changes in the distribution and structure of forested land, particularly near the outer perimeter of municipal boundaries. It is important that these associations between local Lyme disease risk and landscape configuration are explored in settings where tick populations are established, through active surveillance to understand the impacts that neighbourhood development may have on entomological hazard and exposure risk through longitudinal evaluations.

A notable strength of our study is that we assessed the association between physical area and observed Lyme disease incidence at the neighbourhood scale–which may better reflect specific impacts from human alteration of the landscape. The neighbourhood scale also represents a suitable population size for cost-effective and targeted health intervention campaigns. Furthermore, this study leveraged recent PlanetScope satellite imagery to classify land cover and produce current estimates of local forested area. PlanetScope images have a much higher spatial resolution than ‘off-the-shelf’ land cover data products, which are typically generated from multiple sources and resolutions for wider areas with an update schedule of five to ten years. Our results demonstrate that these products are assets for future studies that broaden the land use classifications to include edge contrast types as well as longitudinal image analysis to evaluate local risk over time.

The nature of the available disease surveillance data presented several challenges to our statistical analysis. First, as Lyme disease is an emerging public health concern in the Ottawa region, reported cases are relatively low compared to other regions where transmission cycles of *B*. *burgdorferi* have been active for a longer period. Even aggregated to the neighbourhood scale and across four years, incidence was low (n = 581). In our study, it is lower still because our study focused on cases that occurred–to the patient’s best recollection–at their residence or within the same neighbourhood; nearly 80% of Lyme disease cases in Ottawa suspected their exposure occurred away from their home neighbourhood. In the northeast United States, it is commonly accepted that most tick exposures resulting in Lyme disease occur on residential properties [60], however, there is little evidence as to the true difference in magnitude of risk from residential, recreational, and occupational exposures [58]. In contrast, recent intervention control studies on residential properties [61, 62] found no reduction in tick encounters or tick-borne disease incidence, suggesting that exposures occurring beyond the residential yard are more frequent than expected. While it is possible that misclassification resulting from self-report of the ‘most likely’ location impacts the case totals for some neighbourhoods, most Lyme disease cases (80%) reported to Ottawa Public Health specified only one location despite the opportunity to identify up to five. It is possible that recall bias may differ across neighbourhood types (i.e., urban, suburban, rural), wherein suburban and rural residents have different levels of perceived risk from ticks on their own property while those who have a lower perception of property-level risk might seek to explain their encounters through recreational activities that occurred off-property; such heterogeneity in attitudes could produce biased parameter estimates. Another possibility is that residents of Ottawa are more at risk of recreational exposures compared to other municipalities due to the proximity and integration of the Greenbelt across neighbourhoods. Furthermore, tick populations are still emerging in Ottawa and are more likely to be uneven in distribution compared to regions in the United States where prevailing exposure locations are residential [17]. However, by aggregating to the level of neighbourhood, rather than assigning exposure to reported point locations, we mitigated the effect of patient recall error commonly associated with tick bite encounters [63]. This also aligned our analysis to a scale relevant for different Lyme disease interventions [64]. Additional studies that perform active tick surveillance on residential properties and neighbourhoods across the continuum of developed, herbaceous and woodlands land cover types would help increase our understanding of whether property-level observations of *B*. *burgdorferi*-infected host-seeking ticks follow patterns of Lyme disease outcomes reported to public health officials.

While Ottawa’s status as an urban centre with emerging Lyme disease risk makes it a prime location for this analysis, investigations at the local scale in additional, similar municipalities would further illuminate the true relationship between neighbourhood landscape composition and residential Lyme disease risk. As municipalities plan for future growth while simultaneously responding to climate change, land development guidelines that consider public health impacts are required. While this study is most relevant to Lyme disease at present, the local effects observed may translate to the risk of other emerging tick-borne diseases (e.g., anaplasmosis, *B*. *miyamotoi*, alpha-gal syndrome, ehrlichiosis, babesiosis) considering the expanding range of additional tick species typically found only in southern latitudes [65, 66].

This research quantifies aspects of neighbourhood forests that are important in the context of residential Lyme disease risk in areas of tick habitat expansion. Specifically, these findings highlight significant interactions between neighbourhood-level landscape characteristics–a scale that better reflects the characteristics of local communities–that should be explored further within the context of landscape development and planning guidelines. Although the hypothesized model including interaction with the socio-economic factor did not perform well, the presence of a statistically significant interaction encourages further research. The methods and results are relevant to similar urban areas experiencing outward expansion during a period of Lyme disease emergence. Our findings also reinforce recent studies that emphasize the importance of geographic scale for selection of appropriate types of Lyme disease interventions [64], including educational campaigns aimed at improving the behaviours and practices of residents that may make them otherwise vulnerable. Lastly, by characterizing neighbourhoods through socio-economic and landscape factors, this project has established a basis for future research and predictive mapping efforts for residential areas threatened by invading ticks and emergent Lyme disease.

## Supporting information

S1 Table Lyme disease case demographics by primary exposure location classification, 2017–2020. (DOCX)

S2 Table Moran’s *I* and associated *P* values for tests of spatial autocorrelation using a Queen’s case contiguity weight matrix and Monte Carlo simulations. Tests examined similarity of residential Lyme disease (LD) cases and model residuals following negative binomial regression analyses between bordering neighbourhoods.
(DOCX)

S3 Table Incidence rate ratios and 95% confidence intervals for aspatial negative binomial (NB) multivariable generalized linear models of hypothesized neighbourhood characteristic relationships including interaction terms. (DOCX)

S1 Fig Pairwise correlation matrix between all independent variables explored through hypothesized relationships. (TIF)

S1 File Listing of data sets and sources. (DOCX)
