# Supplementary material for: Risk factors for Lyme disease resulting from residential exposure amidst emerging Ixodes scapularis populations: A neighbourhood-level analysis of Ottawa, Ontario
Source: PLoS One. 2023 Aug 24;18(8):e0290463. doi: 10.1371/journal.pone.0290463 (PMC10449184; doi:10.1371/journal.pone.0290463)
Supplement: S1 Table — (DOCX) [file pone.0290463.s001.docx]

**S1 Table.** Lyme disease case demographics by primary exposure location classification, 2017-2020

|  | All cases  (n = 581) | All cases with exposure data  (n = 534) | Travel*^a^* exposures  (n = 343) | Residential*^b^* exposures  (n = 118) | Non-residential*^c^*, Ottawa exposures (n = 73) |
| --- | --- | --- | --- | --- | --- |
|  | *n* (%) | *n* (%) | *n* (%) | *n* (%) | *n* (%) |
| Mean Age ± SD | 47.98 ± 41.96 | 48.55 ± 43.34 | 50.80 ± 51.41 | 46.43 ± 22.65 | 41.42 ± 20.86 |
| Age Group |  | |  | |  |
| < 14 | 80 (13.8) | 73 (13.7) | 42 (12.2) | 20 (16.9) | 11 (15.1) |
| 15 – 24 | 30 (5.2) | 26 (4.9) | 14 (4.1) | 3 (2.5) | 9 (12.3) |
| 25 – 34 | 48 (8.3) | 44 (8.2) | 27 (7.9) | 10 (8.5) | 7 (9.6) |
| 35 – 44 | 72 (12.4) | 64 (12.0) | 39 (11.4) | 16 (13.6) | 9 (12.3) |
| 45 – 54 | 105 (18.1) | 93 (17.4) | 63 (18.4) | 20 (16.9) | 10 (13.7) |
| 55 – 64 | 125 (21.5) | 119 (22.3) | 81 (23.6) | 20 (16.9) | 18 (24.7) |
| 65+ | 121 (20.8) | 115 (21.5) | 77 (22.4) | 29 (24.6) | 9 (12.3) |
| Gender |  |  |  |  |  |
| Male | 350 (60.2) | 323 (60.5) | 214 (62.4) | 67 (56.8) | 42 (57.5) |
| Female | 231 (39.8) | 211 (39.5) | 129 (37.6) | 51 (43.2) | 31 (42.5) |
| Neighbourhood Type*^d^* |  |  |  |  |  |
| Urban | 176 (30.3) | 165 (30.9) | 129 (37.6) | 12 (10.2) | 24 (32.9) |
| Suburban | 286 (49.2) | 262 (49.1) | 173 (50.4) | 49 (41.5) | 40 (54.8) |
| Rural | 116 (20.0) | 104 (19.5) | 38 (11.1) | 57 (48.3) | 9 (12.3) |

*^a^* Travel determined by location of primary (most likely) exposure location if more than one exposure location was named by the case.
*^b^* Residential exposure status determined by location of primary (most likely) exposure location, if more than one exposure location was named by the case, and matching that location to the neighbourhood of the home address.
*^c^* Non-residential cases are all cases that were not travel-related, as determined by location of primary (most likely) exposure location, who were not identified as being exposed at/near their home address.
*^d^* Individuals who provided no address information are omitted from neighbourhood type totals and proportions.
