# Supplementary material for: Risk factors for Lyme disease resulting from residential exposure amidst emerging Ixodes scapularis populations: A neighbourhood-level analysis of Ottawa, Ontario
Source: PLoS One. 2023 Aug 24;18(8):e0290463. doi: 10.1371/journal.pone.0290463 (PMC10449184; doi:10.1371/journal.pone.0290463)
Supplement: S2 Table — Tests examined similarity of residential Lyme disease (LD) cases and model residuals following negative binomial regression analyses between bordering neighbourhoods. (DOCX) [file pone.0290463.s002.docx]

**S2 Table.** Moran’s *I* and associated *P* values for tests of spatial autocorrelation using a Queen’s case contiguity weight matrix and Monte Carlo simulations. Tests examined similarity of residential Lyme disease (LD) cases and model residuals following negative binomial regression analyses between bordering neighbourhoods.

|  | Aspatial NB regression model | NB regression model with spatial filtering |
| --- | --- | --- |
|  | *Moran’s I* (*P*) | *Moran’s I* (*P*) |
| Residential LD cases | 0.33 (0.001) |  |
| Model 1 | 0.02 (0.32) | 0.002 (0.43) |
| Model 2 | 0.07 (0.13) | 0.05 (0.19) |
| Model 3 | 0.03 (0.26) | -0.045 (0.66) |
| Model 4 | 0.08 (0.09) | -0.012 (0.48) |
| Model 5 | 0.19 (0.007) | 0.11 (0.02) |
