# Supplementary material for: Risk factors for Lyme disease resulting from residential exposure amidst emerging Ixodes scapularis populations: A neighbourhood-level analysis of Ottawa, Ontario
Source: PLoS One. 2023 Aug 24;18(8):e0290463. doi: 10.1371/journal.pone.0290463 (PMC10449184; doi:10.1371/journal.pone.0290463)
Supplement: S3 Table — (DOCX) [file pone.0290463.s003.docx]

**S3 Table.** Incidence rate ratios and 95% confidence intervals for aspatial negative binomial (NB) multivariable generalized linear models of hypothesized neighbourhood characteristic relationships including interaction terms.

|  |  | *Aspatial NB model* | | |
| --- | --- | --- | --- | --- |
| **Model** | **Variables*^a^*** | IRR  (95% CI) | *P* |  |
| 1 | Proportion forested land cover | 1.47 (0.97, 2.21) | 0.05 |  |
|  | Mean forest patch size | 5.04 (2.37, 11.39) | < 0.001 |  |
|  | Moran eigenvector filter | n/a | n/a |  |
|  | Proportion forest x Forest patch size | 0.62 (0.47, 0.80) | < 0.001 |  |
|  | AIC | 262.83 | | |
| 2 | Forest patches (> 0.01 km^2^) | 1.00 (0.43, 2.44) | 0.99 |  |
|  | Edge-to-area ratio | 0.50 (0.30, 0.83) | 0.008 |  |
|  | Moran eigenvector filter | n/a | n/a |  |
|  | Forest patches x Edge-to-area ratio | 0.51 (0.22, 1.21) | 0.136 |  |
|  | AIC | 272.83 | | |
| 3 | Forest patches (> 0.01 km^2^) | 1.80 (1.18, 2.82) | < 0.001 |  |
|  | Mean forest patch size | 2.30 (1.26, 4.61) | 0.0012 |  |
|  | Moran eigenvector filter | n/a | n/a |  |
|  | Forest patches x Forest patch size | 0.79 (0.51, 1.23) | 0.203 |  |
|  | AIC | 269.56 | | |
| 4 | Mean forest patch size | 24.27 (5.63, 118.91) | < 0.001 |  |
|  | Edge-to-area ratio | 1.59 (0.84, 3.08) | 0.116 |  |
|  | Moran eigenvector filter | n/a | n/a |  |
|  | Forest patch size x Edge-to-area ratio | 6.43 (2.20, 19.59) | < 0.001 |  |
|  | AIC | 265.21 | | |
| 5 | Median household income ($000) | 1.47 (0.96, 2.24) | 0.049 |  |
|  | Edge-to-area ratio | 0.45 (0.30, 0.66) | < 0.001 |  |
|  | Moran eigenvector filter | n/a | n/a |  |
|  | Median household income x Edge-to-area ratio | 0.63 (0.40, 0.98) | 0.038 |  |
|  | AIC | 280.99 | | |
