# Supplementary figures and images for: Risk factors for Lyme disease resulting from residential exposure amidst emerging Ixodes scapularis populations: A neighbourhood-level analysis of Ottawa, Ontario
Source: PLoS One. 2023 Aug 24;18(8):e0290463. doi: 10.1371/journal.pone.0290463 (PMC10449184; doi:10.1371/journal.pone.0290463)

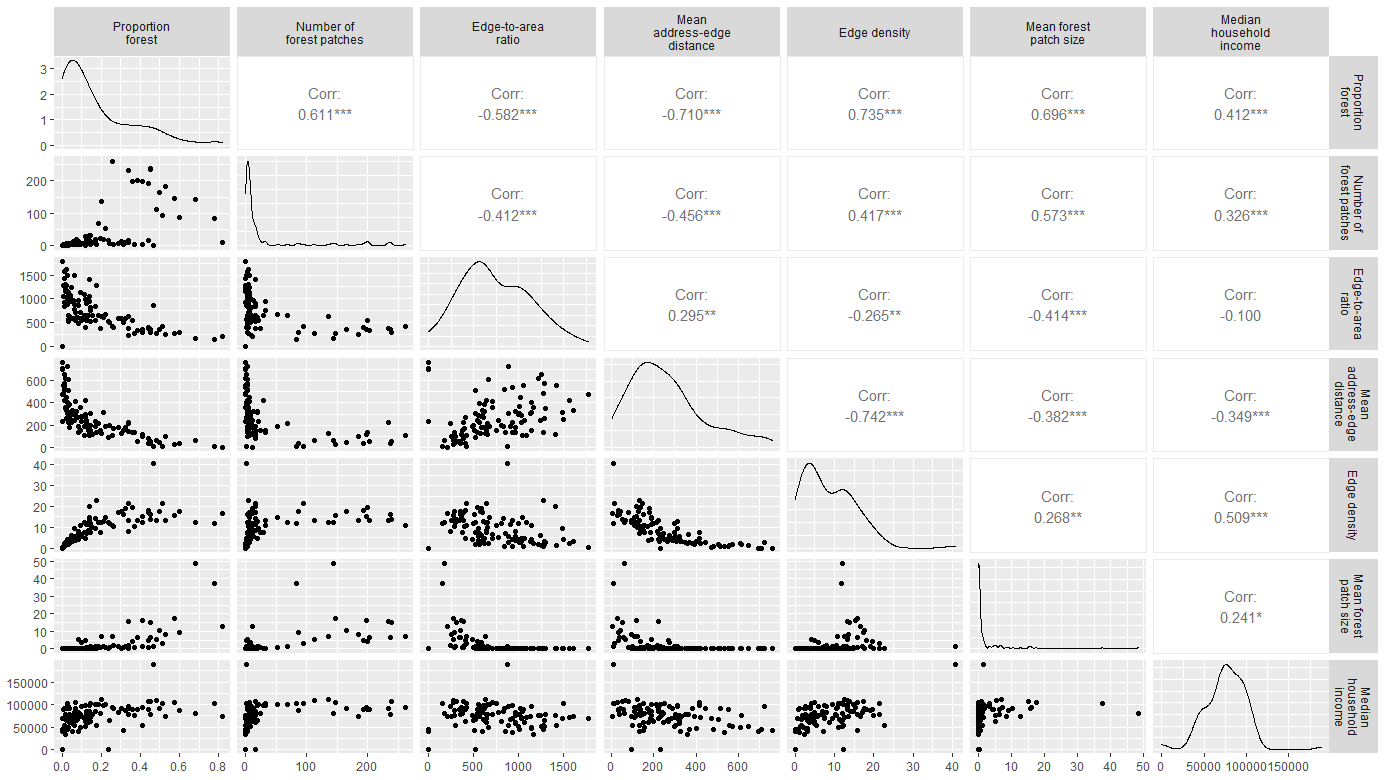

Supplement: S1 Fig — (TIF) [file pone.0290463.s004.tif]
