# Supplementary material for: Risk factors for Lyme disease resulting from residential exposure amidst emerging Ixodes scapularis populations: A neighbourhood-level analysis of Ottawa, Ontario
Source: PLoS One. 2023 Aug 24;18(8):e0290463. doi: 10.1371/journal.pone.0290463 (PMC10449184; doi:10.1371/journal.pone.0290463)
Supplement: S1 File — (DOCX) [file pone.0290463.s005.docx]

**Listing of data sources**

| **Data** Description | **Date range** | **Data format** | **Source** | **URL** | **Licence** |
| --- | --- | --- | --- | --- | --- |
| **Lyme disease surveillance data**  Annual surveillance data of Lyme disease cases reported to Ottawa Public Health, including age, sex, date of disease onset, address, and five locations identified as the site of suspected tick exposure | 2017-2020 | Table, CSV | Public Health Information System (iPHIS) of Public Health Ontario | [Ottawa Public Health](https://www.ottawapublichealth.ca/en/reports-research-and-statistics/infectious-diseases.aspx) | Data Sharing Agreement |
| **Neighbourhood boundaries**  Boundaries created by the Ottawa Neighbourhood study to analyse population statistics. | November 25, 2019 | Polygon  shapefile (.SHP) | Ottawa Neighbourhood Study (ONS) | [Open Data Ottawa](https://open.ottawa.ca/datasets/ottawa::ottawa-neighbourhood-study-ons-neighbourhood-boundaries-gen-2/about) | [Open Government Licence - Canada v2.0](https://open.ottawa.ca/pages/open-data-licence) |
| **Neighbourhood statistics of social determinants**  Socioeconomic and demographic variables selected from the Statistics Canada census and calculated within ONS-delineated boundaries (e.g., median household income, population estimates) | 2016 | with above | Statistics Canada, ONS | [Ottawa Neighbourhood Study](https://www.neighbourhoodstudy.ca/) | [*Statistics Canada Open Licence*](https://www.statcan.gc.ca/en/reference/licence) |
| **Land Imagery** 38 images selected from daily 4-band multispectral satellite data and mosaicked into one high-resolution image of Ottawa.  The land classification generated from a mosaic of PlanetScope satellite imagery | August 2018 | TIF  3.7 m resolution | PlanetScope | Website of imagery products:  [Planet Labs PBC](https://www.planet.com/products/monitoring/)  Classification:  [uOttawa Dataverse](https://borealisdata.ca/dataset.xhtml?persistentId=doi:10.5683/SP3/IFS9UE) | [Education and Research program End User Licence Agreement](https://www.planet.com/markets/education-and-research/)  CC BY 4.0 |
